# Nested Grassmannians for Dimensionality Reduction with Applications

**Published:** 2022-03

**Authors:** Chun-Hao Yang, Baba C. Vemuri

**Affiliations:** Institute of Applied Mathematical Science, National Taiwan University, Taipei, Taiwan; Department of CISE, University of Florida, Gainesville, FL, USA

**Keywords:** Grassmann Manifolds, Dimensionality Reduction, Shape Analysis, Homogeneous Riemannian Manifolds

## Abstract

In the recent past, nested structures in Riemannian manifolds has been studied in the context of dimensionality reduction as an alternative to the popular principal geodesic analysis (PGA) technique, for example, the principal nested spheres. In this paper, we propose a novel framework for constructing a nested sequence of homogeneous Riemannian manifolds. Common examples of homogeneous Riemannian manifolds include the *n*-sphere, the Stiefel manifold, the Grassmann manifold and many others. In particular, we focus on applying the proposed framework to the Grassmann manifold, giving rise to the nested Grassmannians (NG). An important application in which Grassmann manifolds are encountered is planar shape analysis. Specifically, each planar (2D) shape can be represented as a point in the complex projective space which is a complex Grassmann manifold. Some salient features of our framework are: (i) it explicitly exploits the geometry of the homogeneous Riemannian manifolds and (ii) the nested lower-dimensional submanifolds need not be geodesic. With the proposed NG structure, we develop algorithms for the supervised and unsupervised dimensionality reduction problems respectively. The proposed algorithms are compared with PGA via simulation studies and real data experiments and are shown to achieve a higher ratio of expressed variance compared to PGA.

## 1. Introduction

Riemannian manifolds are often used to model the sample space in which features derived from the raw data encountered in many medical imaging applications live. Common examples include the diffusion tensors (DTs) in diffusion tensor imaging (DTI) (Basser et al., 1994), the ensemble average propagator (EAP) (Callaghan, 1993). Both DTs and EAP are used to capture the diffusional properties of water molecules in the central nervous system by non-invasively imaging the tissue via diffusion weighted magnetic resonance imaging. In DTI, diffusion at a voxel is captured by a DT, which is a 3 × 3 symmetric positive-definite matrix, whereas EAP is a probability distribution characterizing the local diffusion at a voxel, which can be parametrized as a point on the Hilbert sphere. Another example is the shape space used to represent shapes in shape analysis. There are many ways to represent a shape, and the most simple one is to use landmarks. For the landmark-based representation, the shape space is called Kendall’s shape space (Kendall, 1984). Kendall’s shape space is in general a stratified space (Goresky and MacPherson, 1988; Feragen et al., 2014), but for the special case of planar shapes, the shape space is the complex projective space, which is a complex Grassmann manifold. The examples mentioned above are often high-dimensional: a DTI scan usually contains half a million DTs; the shape of the Corpus Callosum (which is used in our experiments) is represented by a several hundreds of boundary points in ${\mathbb{R}}^{2}$. Thus, in these cases, dimension reduction techniques, if applied appropriately, can benefit the subsequent statistical analysis.

For data on Riemannian manifolds, the most widely used dimensionality reduction method is the principal geodesic analysis (PGA) (Fletcher et al., 2004), which generalizes the principal component analysis (PCA) to manifold-valued data. In fact, there are many variants of PGA. Fletcher et al. (2004) proposed to find the geodesic submanifold of a certain dimension that maximizes the projected variance and computationally, it was achieved via a linear approximation, i.e., applying PCA on the tangent space at the intrinsic mean. This is sometimes referred to as the tangent PCA. Note that this approximation requires the data to be clustered around the intrinsic mean, otherwise the tangent space approximation to the manifold leads to inaccuracies. Later on, Sommer et al. (2010) proposed the Exact PGA (EPGA), which does not use any linear approximation. However, EPGA is computationally expensive as it requires two non-linear optimizations steps per iteration (projection to the geodesic submanifold and finding the new geodesic direction such that the loss of information is minimized). Chakraborty et al. (2016) partially solved this problem for manifolds with constant sectional curvature (spheres and hyperbolic spaces) by deriving closed form formulae for the projection. Other variants of PGA include but are not limited to sparse exact PGA (Banerjee et al., 2017), geodesic PCA (Huckemann et al., 2010), and probabilistic PGA (Zhang and Fletcher, 2013). All these methods focus on projecting data to a *geodesic submanifold* as in PCA where one projects data to a vector subspace. Instead, one can also project data to a submanifold that minimizes the reconstruction error without any further restrictions, e.g. being geodesic. This is the generalization of the principal curve (Hastie and Stuetzle, 1989) to Riemannian manifolds presented in Hauberg (2016).

Another feature of PCA is that it produces a sequence of nested vector subspaces. From this observation, Jung et al. (2012) proposed the *principal nested spheres* (PNS) by embedding an *n* — 1-sphere into an *n*-sphere, and the embedding is not necessarily isometric. Hence PNS is more general than PGA in that PNS is not required to be geodesic. Similarly, for the manifold *P*_*n*_ of *n* × *n* SPD matrices, Harandi et al. (2018) proposed a geometry-aware dimension reduction by projecting data in *P*_*n*_ to *P*_*m*_ for some *m* ≪ *n*. They also applied the nested *P*^*n*^ for the supervised dimensionality reduction problem. Damon and Marron (2014) considered a nested sequence of relations which determine a nested sequence of submanifolds that are not necessarily geodesic. They showed various examples, including Euclidean space and the *n*-sphere, depicting how the nested relations generalized PCA and PNS. However, for an arbitrary Riemannian manifold, it is not clear how to construct a nested submanifold. Another generalization of PGA was proposed by Pennec et al. (2018), called the exponential barycentric subspace (EBS). A *k*-dimensional EBS is defined as the locus of weighted exponential barycenters of (*k* + 1) affinely independent reference points. The EBSs are naturally nested by removing or adding reference points.

Unlike PGA which can be applied to any Riemannian manifolds, the construction of the nested manifolds relies heavily on the geometry of the specific manifold, and there is no general principle for such a construction. All the examples described above (spheres and *P*_*n*_) and several others such as the Grassmannian, Stiefel etc. are homogeneous Riemannian manifolds (Helgason, 1979), which intuitively means that all points on the manifold ‘look’ the same. In this work, we propose a general framework for constructing a nested sequence of homogeneous Riemannian manifolds, and, via some simple algebraic computation, show that the nested sphere and the nested *P*_*n*_ can indeed be derived within this framework. We will then apply this framework to the Grassmann manifolds, called the nested Grassmann manifolds (NG). The Grassmann manifold Gr(p,V) is the manifold of all *p*-dimensional subspaces of the vector space V where 1≤p≤dim V. Usually $V={\mathbb{R}}^{n}$ or $V={\mathbb{C}}^{n}$. An important example is Kendall’s shape space of 2D shapes. The space of all shapes determined by *k* landmarks in ${\mathbb{R}}^{2}$ is denoted by $\Sigma_{2}^{k}$, and Kendall (1984) showed that it is isomorphic to the complex projective space $\mathbb{C}P^{k-2}≅\text{Gr}\left(1,{\mathbb{C}}^{k-1}\right)$. In many applications, the number *k* of landmarks is large, and so is the dimension of $\text{Gr}\left(1,{\mathbb{C}}^{k-1}\right)$. The core of the proposed dimensionality reduction involves projecting data on Gr(p,V) to $\text{Gr}(p,\tilde{V})$ with $\text{dim }\tilde{V}\ll \text{dim }V$. The main contributions of this work are as follows: (i) We propose a general framework for constructing a nested sequence of homogeneous Riemannian manifolds unifying the recently proposed nested spheres (Jung et al., 2012) and nested SPD manifolds (Harandi et al., 2018). (ii) We present novel dimensionality reduction techniques based on the concept of NG in both supervised and unsupervised settings respectively. (iii) We demonstrate via several simulation studies and real data experiments, that the proposed NG can achieve a higher ratio of expressed variance compared to PGA.

The rest of the paper is organized as follows. In Section 2, we briefly review the definition of homogeneous Riemannian manifolds and present the recipe for the construction of nested homogeneous Riemannian manifolds. In Section 3, we first review the geometry of the Grassmannian. By applying the procedure developed in Section 2, we present the nested Grassmann manifolds representation and discuss some of its properties in details. Then we describe algorithms for our unsupervised and supervised dimensionality reduction techniques, called the Principal Nested Grassmanns (PNG), in Section 4. In Section 5, we present several simulation studies and experimental results showing how the PNG technique performs compared to PGA under different settings. Finally, we draw conclusions in Section 6.

## 2. Nested Homogeneous Spaces

In this section, we introduce the structure of nested homogeneous Riemannian manifolds. A Riemannian manifold (*M*, *τ*) is *homogeneous* if the group of isometries *G* = *I*(*M*) admitted by the manifold acts transitively on *M* (Helgason, 1979), i.e., for *x*, *y* ∈ *M*, there exists g ∈ *G* such that *g*(*x*) = *y*. In this case, *M* can be identified with *G*/*H* where *H* is an isotropy subgroup of *G* at some point *p* ∈ *M* i.e. *H* = {*g* ∈ *G* : *g*(*p*) = *p*}. Examples of homogeneous Riemannian manifolds include but are not limited to, the *n*-spheres *S*^*n*−1^ = SO(*n*)/SO(*n* – 1), the SPD manifolds *P*_*n*_ = GL(*n*)/O(*n*), the Stiefel manifolds St(*m*, *n*) = SO(*n*)/SO(*n* − *m*), and the Grassmann manifolds Gr(*p*, *n*) = SO(*n*)/S(O(*p*) × O(*n* − *p*)).

In this paper, we focus on the case where *G* is either a real or a complex matrix Lie group, i.e. *G* is a subgroup of GL(n,ℝ) or GL(n,ℂ). The main idea behind the construction of nested homogeneous spaces is simple: *augmenting the matrix in G in an appropriate way*. With an embedding of the isometry group *G*, the embedding of the homogeneous space *G*/*H* follows naturally from the quotient structure.

Let *G* and $\tilde{G}$ be two connected Lie groups such that $\text{dim }G<\text{dim }\tilde{G}$ and $\tilde{\iota }:G\to \tilde{G}$ be an embedding. For a closed connected subgroup *H* of *G*, let $\tilde{H}=\tilde{\iota }(H)$. Since $\tilde{\iota }$ is an embedding, $\tilde{H}$ is also a closed subgroup of $\tilde{G}$. Now the canonical embedding of *G*/*H* in $\tilde{G}/\tilde{H}$ is defined by $\iota (gH)=\tilde{\iota }(g)\tilde{H}$ for *g* ∈ *G*. It is easy to see that *ι* is well-defined. Let *g*_1_, *g*_2_ ∈ *G* be such that *g*_1_ = *g*_2_*h* for some *h* ∈ *H*. Then

$$\iota \left(g_{1}H\right)=\tilde{\iota }\left(g_{1}\right)\tilde{H}=\tilde{ı}\left(g_{2}h\right)\tilde{H}=\tilde{\iota }\left(g_{2}\right)\tilde{\iota }(h)\tilde{H}=\tilde{\iota }\left(g_{2}\right)\tilde{H}=\iota \left(g_{2}H\right).$$

Now for the homogeneous Riemannian manifolds (*M* = *G*/*H*, *τ*_1_) and $\left(\tilde{M}=\tilde{G}/\tilde{H},\tau_{2}\right)$, denote the left-*G*-invariant, right-*H*-invariant metric on *G* (resp. left-$\tilde{G}$-invariant, right-$\tilde{H}$-invariant metric on $\tilde{G}$) by ${\bar{\tau }}_{1}$ and ${\bar{\tau }}_{2}$, respectively (see Cheeger and Ebin (1975, Prop. 3.16(4))).

**Proposition 1** *If $\tilde{\iota }:G\to \tilde{G}$ is isometric*, *then so is $\iota :G/H\to \tilde{G}/\tilde{H}$*.

**Proof** Denote the Riemannian submersions by *π* : *G* → *G*/*H* and $\tilde{\pi }:\tilde{G}\to \tilde{G}/\tilde{H}$. Let *X* and *Y* be vector fields on *G*/*H* and $\bar{X}$ and $\bar{Y}$ be their horizontal lifts respectively, i.e. $d\pi (\bar{X})=X$ and $d\pi (\bar{Y})=Y$. By the definition of Riemannian submersions, *dπ* is isometric on the horizontal spaces, i.e. ${\bar{\tau }}_{1}(\bar{X},\bar{Y})=\tau_{1}(d\pi (\bar{X}),d\pi (\bar{Y}))=\tau_{1}(X,Y)$. Since $\tilde{ı}$ is isometric, we have ${\bar{\tau }}_{1}(\bar{X},\bar{Y})={\bar{\tau }}_{2}(d\tilde{\iota }(\bar{X}),d\tilde{\iota }(\bar{Y}))$. By the definition of *ι*, we also have $\iota \circ \pi =\tilde{\pi }\circ \tilde{\iota }$, which implies $d\iota \circ d\pi =d\tilde{\pi }\circ d\tilde{\iota }$. Hence,

$$\tau_{1}(X,Y)={\bar{\tau }}_{1}(\bar{X},\bar{Y})={\bar{\tau }}_{2}(d\tilde{\iota }(\bar{X}),d\tilde{\iota }(\bar{Y}))\;=\tau_{2}((d\tilde{\pi }\circ d\tilde{\iota })(\bar{X}),(d\tilde{\pi }\circ d\tilde{\iota })(\bar{Y}))\;=\tau_{2}((d\iota \circ d\pi )(\bar{X}),(d\iota \circ d\pi )(\bar{Y}))\;=\tau_{2}(d\iota (X),d\iota (Y))$$

where the third equality follows from the isometry of $d\tilde{\pi }$. ■

**Figure 1:**
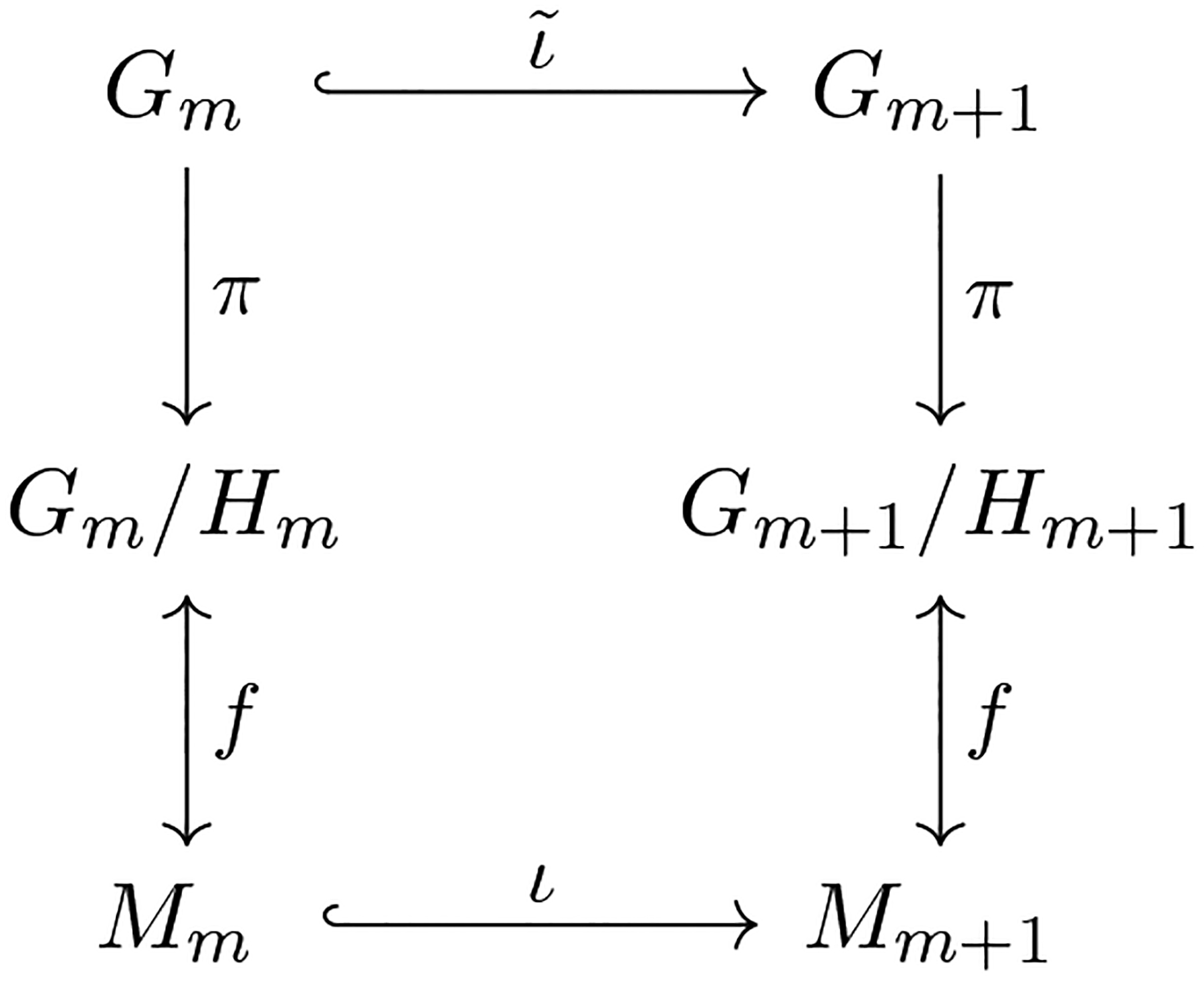
Commutative diagram of the induced embedding for homogeneous spaces.

Proposition 1 simply says that if the isometry group is isometrically embedded, then the associated homogeneous Riemannian manifolds will also be isometrically embedded. Conversely, if we have a Riemannian submersion $\tilde{f}:\tilde{G}\to G$, it can easily be shown that the induced map $f:\tilde{G}/\tilde{H}\to G/H$ would also be a Riemannian submersion where $H=\tilde{f}(\tilde{H})$. The construction above can be applied to a sequence of homogeneous spaces ${\left\{M_{m}\right\}}_{m=1}^{\infty }$, i.e. the embedding *ι*_*m*_ : *M*_*m*_ → *M*_*m*+1_ can be induced from the embedding of the isometry groups ${\tilde{\iota }}_{m}:G_{m}\to G_{m+1}$ where *G*_*m*_ = *I*(*M*_*m*_) provided dim *G*_*i*_ < dim *G*_*j*_ for *i* < *j*. See Figure 1 for the structure of nested homogeneous spaces.

## 3. Nested Grassmann Manifolds

In this section, we will apply the theory of nested homogeneous space from the previous section to the Grassmann manifolds. First, we briefly review the geometry of the Grassmann manifolds in Section 3.1. With the theory in Section 2, we derive the nested Grassmann manifolds in Section 3.2, and the derivation for nested spheres and nested SPD manifolds are carried out in Section 3.3.

### 3.1 The Riemannian Geometry of Grassmann Manifolds

To simplify the notation, we assume $V={\mathbb{R}}^{n}$ and write $\text{Gr}(p,n)≔\text{Gr}\left(p,{\mathbb{R}}^{n}\right)$. All the results presented in this section can be easily extended to the case of $V={\mathbb{C}}^{n}$ by replacing transposition with conjugate transposition and orthogonal groups with unitary groups. The Grassmann manifold Gr(*p*, *n*) is the manifold of all *p*-dimensional subspaces of ${\mathbb{R}}^{n}$, and for a subspace 𝒳 ∈ Gr(*p*, *n*),. we write 𝒳 = span(*X*) where the columns of *X* form an orthonormal basis for 𝒳. The space of all *n* × *p* matrices *X* such that *X*^*T*^ *X* = *I*_*p*_ called the *Stiefel manifold*, denoted by St(*p*, *n*). Special cases of Stiefel manifolds are the Lie group of all orthogonal matrices, O(*n*) = St(*n*, *n*), and the *n*-sphere, *S*^*n*−1^ = St(1, *n*). The Stiefel manifold with the induced Euclidean metric (i.e. for *U*, *V* ∈ *T*_*X*_St(*p*, *n*). 〈*U*, *V*〉_X_ = tr(*U*^*T*^*V*)) is a homogeneous Riemannian manifold, St(*p*, *n*) = SO(*n*)/SO(*n* − *p*). A canonical Riemannian metric on the Grassmann manifold can be inherited from the metric on St(*p*, *n*) since it is invariant to the left multiplication by elements of *O*(*n*) (Absil et al., 2004; Edelman et al., 1998). The Grassmann manifold with this metric is also homogeneous, Gr(*p*, *n*) = SO(*n*)/*S*(O(*p*) × O(*n* − *p*)).

With this canonical metric on the Grassmann manifolds, the geodesic can be expressed in closed form. Let 𝒳 = span(*X*) ∈ Gr(*p*, *n*) where *X* ∈ St(*p*, *n*) and *H* be an *n* × *p* matrix. Then the geodesic *γ*(*t*) with *γ*(0) = 𝒳 and *γ*′(0) = *H* given by *γ*_𝒳,H_(*t*) = span(*XV* cos Σ*t* + *U* sin Σ*t*) where *H* = *U*Σ*V*^*T*^ is the compact singular value decomposition (Edelman et al., 1998, Theorem 2.3). The *exponential map* at 𝒳 is a map from T_𝒳_ Gr(*p*, *n*) to Gr(*p*, *n*) defined by Exp_𝒳_*H* = *γ*_𝒳,H_ (1) = span(*XV* cos Σ + *U* sin Σ). If *X*^*T*^*Y* is invertible, the geodesic distance between 𝒳 = span(*X*) and 𝒴 = span(*Y*) is given by $d_{g}^{2}(𝒳,𝒴)=\text{tr }\Theta^{2}=\sum_{i=1}^{p}\theta_{i}^{2}$ where (I − *XX*^*T*^)*Y* (*X*^*T*^*Y* )^−1^ = *U*Σ*V*^*T*^, *U* ∈ St(*p*, *n*), V ∈ O(*p*), and Θ = tan^−1^ Σ. The diagonal entries *θ*_1_, …, *θ*_*k*_ of Θ are known as the principal angles between 𝒳 and 𝒴.

### 3.2 Embedding of Gr(*p*, *m*) in Gr(*p*, *n*)

Let 𝒳 = span(*X*) ∈ Gr(*p*, *m*), *X* ∈ St(*p*, *m*). The map *ι* : Gr(*p*, *m*) → Gr(*p*, *n*), for *m* < *n*, defined by

$$\iota (𝒳)=\text{span}\left(\left[\begin{array}{c} X\\ 0_{(n-m)\times p} \end{array}\right]\right)$$

is an embedding and it is easy to check that this embedding is isometric (Ye and Lim, 2016, Eq. (8)). However, for the dimensionality reduction problem, the above embedding is insufficient as it is not flexible enough to encompass other possible embeddings. To design flexible embeddings, we apply the framework proposed in Section 2. We consider *M*_*m*_ = Gr(*p*, *m*) for which the isometry groups are *G*_*m*_ = SO(*m*) and *H*_*m*_ = S(O(*p*) × O(*m* − *p*)).

In this paper, we consider the embedding ${\tilde{\iota }}_{m}:\text{SO}(m)\to \text{SO}(m+1)$ given by,

(1) $${\tilde{\iota }}_{m}(O)=\text{GS}\left(R\left[\begin{array}{cc} O & a\\ b^{T} & c \end{array}\right]\right)$$

where *O* ∈ SO(*m*), *R* ∈ SO(*m* + 1), *a*, $b\in {\mathbb{R}}^{m}$, c∈ℝ, *c* ≠ *b*^*T*^*O*^−1^*a*, and GS(·) is the Gram-Schmidt process. Since the Riemannian submersion *π* : SO(*m*) → Gr(*p*, *m*) is defined by *π* (*O*) = span(*O*_p_) where O ∈ SO(*m*) and *O*_*p*_ is the *m* × *p* matrix containing the first *p* columns of *O*, the induced embedding *ι*_*m*_ : Gr(*p*, *m*) → Gr(*p*, *m* + 1) is given by,

$$\iota_{m}(𝒳)=\text{span}\left(R\left[\begin{array}{c} X\\ b^{T} \end{array}\right]\right)=\text{span}\left(\tilde{R}X+vb^{T}\right),$$

where $b\in {\mathbb{R}}^{p}$, *R* ∈ SO(*m* + 1), $\tilde{R}$ contains the first *m* columns of *R* (which means $\tilde{R}\in \text{St}\left(m,m+1\right)$, *υ* is the last column of *R*, and 𝒳 = span(*X*) ∈ Gr(*p*, *m*). It is easy to see that for *R* = *I* and *b* = 0, this gives the natural embedding described in Ye and Lim (2016) and at the beginning of this section.

**Proposition 2** *If b* = 0, *then ι*_*m*_ *is an isometric embedding*.

**Proof** With Proposition 1, it suffices to show that ${\tilde{\iota }}_{m}$ is isometric when *b* = 0. Note that as *ι*_*m*_ is independent of *a* and *c* in the definition of ${\tilde{\iota }}_{m}$, we can assume *a* = 0 and *c* = 1 without loss of generality. If ${\tilde{\iota }}_{m}$ simplifies to

$${\tilde{\iota }}_{m}(O)=R\left[\begin{array}{ll} O & 0\\ 0 & 1 \end{array}\right]$$

where *R* ∈ SO(*m* + 1). The Riemannian distance on SO(*n*) given the induced Euclidean metric is $d_{\text{SO}}\left(O_{1},O_{2}\right)=\frac{1}{\sqrt{2}}{\left\|\text{log }O_{1}^{T}O_{2}\right\|}_{F}$. Then for *O*_1_, *O*_2_ ∈ SO(*m*),

$$d_{\text{SO}}\left({\tilde{\iota }}_{m}\left(O_{1}\right),{\tilde{\iota }}_{m}\left(O_{2}\right)\right)=\frac{1}{\sqrt{2}}{\left\|\text{log}\left(\left[\begin{array}{cc} O_{1}^{T}O_{2} & 0\\ 0 & 1 \end{array}\right]\right)\right\|}_{F}=d_{\text{SO}}\left(O_{1},O_{2}\right).$$

Therefore ${\tilde{\iota }}_{m}$ is an isometric embedding, and so is *ι*_*m*_ by Proposition 1. ■

With the embedding *ι*_*m*_, we can construct the corresponding projection *π*_*m*_ : *Gr*(*p*, *m* + 1) → *Gr*(*p*, *m*) using the following proposition.

**Proposition 3** *The projection π*_*m*_ : *Gr*(*p*, *m*+1) → *Gr*(*p*, *m*) *corresponding to $\iota_{m}(𝒳)=\text{span}\left(\tilde{R}X+vb^{T}\right)$ is given by $\pi_{m}(𝒳)=\text{span}\left({\tilde{R}}^{T}X\right)$*.

**Proof** First, let 𝒴 = span(*Y*) ∈ Gr(*p*, *m*) and 𝒳 = span(*X*) 2 Gr(*p*, *m* + 1) be such that $𝒳=\text{span}\left(\tilde{R}Y+vb^{T}\right)$. Then $XL=\tilde{R}Y+vb^{T}$ for some *L* ∈ GL(*p*). Therefore, $Y={\tilde{R}}^{T}\left(XL-vb^{T}\right)={\tilde{R}}^{T}XL$ and $𝒴=\text{span}(Y)=\text{span}\left({\tilde{R}}^{T}XL\right)=\text{span}\left({\tilde{R}}^{T}X\right)$. Hence, the projection is given by $\pi_{m}(𝒳)=\text{span}\left({\tilde{R}}^{T}X\right)$. This completes the proof. ■

Note that for 𝒳 = span(*X*) 2 Gr(*p*, *m*+1), *ι*_*m*_(*π*_*m*_(𝒳)) = span(*RR*^*T*^ *X*+*vb*^*T*^) = span((*I* − *vv*^*T*^ )*X* +*vb*^*T*^) where $v\in {\mathbb{R}}^{m+1}$ and ∥*υ*∥ = 1. The nested relation can be extended inductively and we refer to this construction as the *nested Grassmann structure*:

$$\text{Gr}(p,m)\overset{\iota_{m}}{↪}\text{Gr}(p,m+1)\overset{\iota_{m+1}}{↪}\;\ldots \;\overset{\iota_{n-2}}{↪}\text{Gr}(p,n-1)\overset{\iota_{n-1}}{↪}\text{Gr}(p,n).$$

Thus the embedding from Gr(*p*, *m*) into Gr(*p*, *n*) can be constructed inductively by *ι* ≔ *ι*_*n*−1_ ∘… ∘*ι*_*m*−1_ ∘ *ι*_*m*_ and similarly for the corresponding projection. The explicit forms of the embedding and the projection are given in the following proposition.

**Proposition 4** *The embedding of Gr*(*p*, *m*) *into Gr*(*p*, *n*) *for m* < *n is given by ι*_A,B_(𝒳) = span(*AX* + *B*) *where A* ∈ *St*(*m*, *n*) *and $B\in {\mathbb{R}}^{n\times p}$ such that A*^*T*^ *B* = 0*. The corresponding projection from Gr*(*p*, *n*) *to Gr*(*p*, *m*) *is given by π*_*A*_ = span(*A*^*T*^ *X*).

**Proof** By the definition, *ι* ≔ *ι*_*n*−1_ ∘…∘*ι*_*m*−1_ ∘*ι*_*m*_ and thus the embedding *ι* : Gr(*p*, *m*) → Gr(*p*, *n*) can be simplified as

$$\iota_{A,B}(𝒳)=\text{span}\left(\left(\prod_{i=m}^{n-1}R_{i}\right)X+\sum_{i=m}^{n-1}\left(\prod_{j=i+1}^{n-1}R_{j}\right)v_{i}b_{i}^{T}\right)=\text{span}(AX+B)$$

where *R*_*i*_ ∈ St(*i*, *i* + 1), *v*_*i*_ is such that [*R*_*i*_ *υ*_*i*_] ∈ O(*i* + 1), $b_{i}\in {\mathbb{R}}^{p}$, *A* = *R*_*n*−1_*R*_*n*−2_ ⋯ *R*_*m*_ ∈ St(*m*, *n*), and $B=\sum_{i=m}^{n-1}\left(\prod_{j=i+1}^{n-1}R_{j}\right)v_{i}b_{i}^{T}$ is an *n* × *p* matrix. Similar to Proposition 3, the projection *π*_*A*_ : Gr(*p*, *n*) → Gr(*p*, *m*) is then given by *π*_*A*_(𝒳) = span(*A*^*T*^*X*). This completes the proof. ■

**Figure 2:**
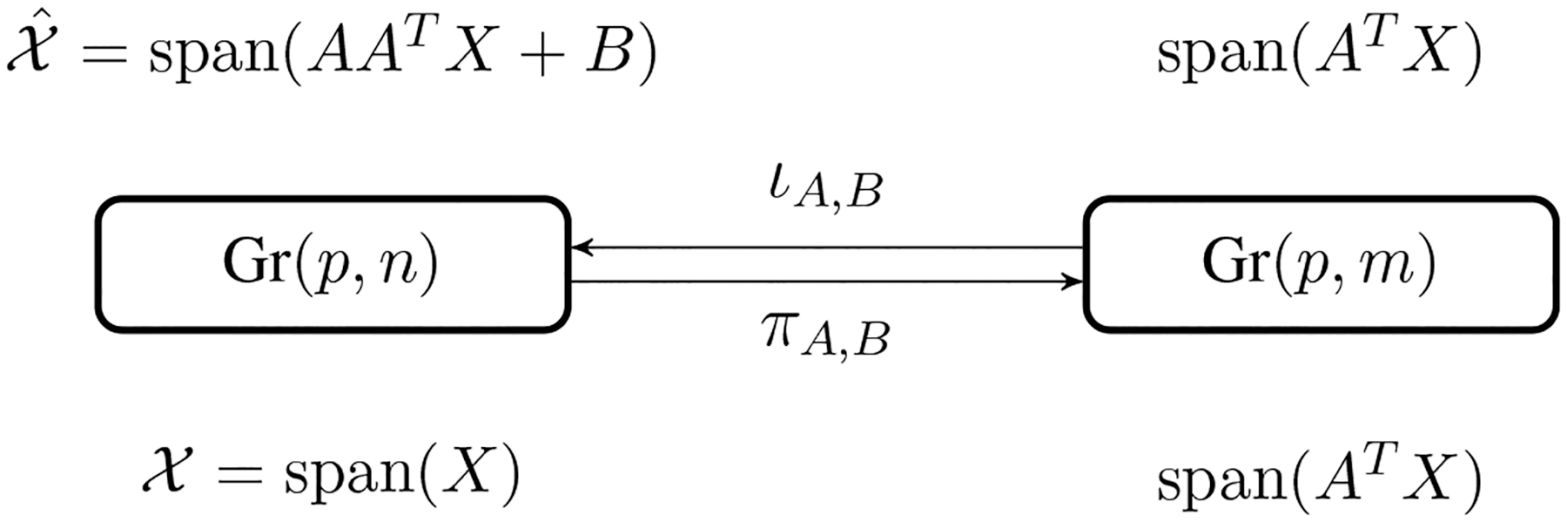
Illustration of the embedding of Gr(*p*, *m*) in Gr(*p*, *n*) parametrized by *A* ∈ St(*m*, *n*) and $B\in {\mathbb{R}}^{n\times p}$ such that *A*^*T*^*B* = 0.

From Proposition 2, if *B* = 0 then *ι*_*A*_ is an isometric embedding. *Hence*, *our nested Grassmann structure is more flexible than PGA as it allows one to project the data onto a non-geodesic submanifold*. An illustration is shown in Figure 2. The results discussed in this subsection can be generalized to any homogeneous space in principle. For a given homogeneous space, once the embedding of the groups of isometries (e.g., Eq. (1)) is determined, the induced embedding and the corresponding projection can be derived akin to the case of Grassmann manifolds.

### 3.3 Connections to Other Nested Structures

The nested homogeneous spaces proposed in this work (see Figure 1) actually provides a unified framework within which, the nested spheres (Jung et al., 2012) and the nested SPD manifolds (Harandi et al., 2018) are special cases.

#### The *n*-Sphere Example:

Since the *n*-sphere can be identified with a homogeneous space *S*^*n*−1^ ≅ O(*n*)/O(*n* − 1), with the embedding (1), the induced embedding of *S*^*n*−1^ into *S*^*n*^ is

$$\iota (x)=\text{GS}\left(R\left[\begin{array}{l} x\\ b \end{array}\right]\right)=\frac{1}{\sqrt{1+b^{2}}}R\left[\begin{array}{l} x\\ b \end{array}\right]=R\left[\begin{array}{c} \text{sin}(r)x\\ \text{cos}(r) \end{array}\right]$$

where *x* ∈ *S*^*n*−1^, b∈ℝ, and $r={\text{cos}}^{-1}\left(\frac{b}{\sqrt{1+b^{2}}}\right)$. This is precisely the nested sphere proposed in Jung et al. (2012, Eq. (2)).

#### The SPD Manifold Example:

For the *m*-dimensional SPD manifold denoted by *P*_*m*_, *G*_*m*_ = GL(*m*) and *H*_*m*_ = O(*m*). Consider the embedding ${\tilde{\iota }}_{m}:\text{GL}(m)\to \text{GL}(m+1)$ given by

$$\tilde{A}={\tilde{\iota }}_{m}(A)=R\left[\begin{array}{cc} A & 0\\ 0 & 1 \end{array}\right]R^{T},$$

where *A* ∈ GL(*m*), *R* ∈ O(*m* + 1) and the corresponding projection ${\tilde{\pi }}_{m}:\text{GL}(m+1)\to \text{GL}(m)$ is

$${\tilde{\pi }}_{m}(\tilde{A})=W^{T}\tilde{A}W$$

where*W* contains the first *m* columns of *R* = [*W υ*] ∈ O(*m* + 1) (i.e., *W* ∈ St(*m*, *m* + 1) and *W*^*T*^ *υ* = 0. The submersion *ψ* ∘ *f* : GL(*m*) → *P*_*m*_ is given by *ψ* ∘ *f*(*A*):*A*^*T*^*A*. Hence the induced embedding *ι*_*m*_ : *P*_*m*_ *→ P*_*m*+1_ and projection *π*_*m* :_ *P*_*m*+1_ → *P*_*m*_ are

$$\iota_{m}\left(X\right)=WXW^{T}+\upsilon \upsilon^{T}\text{ and }\pi_{m}\left(X\right)=W^{T}XW$$

which is the projection map used in Harandi et al. (2018, Eq. (13)). However, Harandi et al. (2018) did not perform any embedding or construct a nested family of SPD manifolds. Recently, it came to our attention that Jaquier and Rozo (2020) derived a similar nested family of SPD manifolds based on the projection maps in Harandi et al. (2018) described above.

## 4. Dimensionality Reduction with Nested Grassmanns

In this section, we discuss how to apply the nested Grassmann structure to the problem of dimension reduction. In Section 4.1 and 4.2, we describe the unsupervised and supervised dimension reduction based on the nested Grassmann manifolds. In Section 4.3, we will discuss the choice of distance metrics required by the dimensionality reduction algorithm and present some technical details regarding the implementation. Analysis of principal nested Grassmann (PNG) will be introduced and discussed in Section 4.4 and Section 4.5.

### 4.1 Unsupervised Dimensionality Reduction

We can now apply the nested Grassmann structure to the problem of unsupervised dimensionality reduction. Suppose that we are given the points, that we seek is obtained by the minimizing the reconstruction error, i.e. 𝒳^1^, …, 𝒳^*N*^ ∈ Gr(*p*, *n*). We would like to have lower dimensional representations in Gr(*p, m*) for 𝒳_1_, …, 𝒳_*N*_ with *m* ≪ *n*. The desired projection map *π*_*A*_ that we seek is obtained by the minimizing the reconstruction error, i.e.

$$L_{u}\left(A,B\right)=\frac{1}{N}\sum_{i=1}^{N}d^{2}\left({𝒳}_{i},{\hat{𝒳}}_{i}\right)=\frac{1}{N}\sum_{i=1}^{N}d^{2}\left(\text{span}\left(X_{i}\right),\text{span}\left(AA^{T}X_{i}+B\right)\right)$$

where *d*(·, ·) is a distance metric on Gr(*p, n*). It is clear that *L*_*u*_ has a O(*m*)-symmetry in the first argument, i.e. *L*_*u*_(*AO*, *B*) = *L*_*u*_(*A, B*) for *O* ∈ *O*(*m*). Hence, the optimization is performed over the space *St*(*m, n*)/*O*(*m*) ≅ *Gr*(*m, n*) when optimizing with respect to this particular loss function. Now we can apply the Riemannian gradient descent algorithm (Edelman et al., 1998) to obtain A and B by optimizing *L*_*u*_(*A, B*) over span(A) ∈ Gr(*m, n*) and $B\in {\mathbb{R}}^{n\times p}$ such that *A*^*T*^*B* = 0. Note that the restriction *A*^*T*^*B* = 0 simply means that the columns of *B* are in the null space of *A*^*T*^, denoted *N*(*A*^*T*^). Hence in practice this restriction can be handled as follows. For arbitrary $\tilde{B}\in {\mathbb{R}}^{n\times p}$ project $\tilde{B}$ on to *N*(*A*^*T*^), i.e. $B=P_{N\left(A^{T}\right)}\tilde{B}$ where $P_{N\left(A^{T}\right)}=I-AA^{T}$ is the projection from ${\mathbb{R}}^{n}$ to *N*(*A*^*T*^). Thus, the loss function can be written as

$$L_{u}\left(A,B\right)=\frac{1}{N}\sum_{i=1}^{N}d^{2}\left(\text{span}\left(X_{i}\right),\text{span}\left(AA^{T}X_{i}+\left(I-AA^{T}\right)B\right)\right)$$

and it is optimized over $\text{Gr}(m,n)\times {\mathbb{R}}^{n\times p}$. Note that since we represent a subspace by its orthonormal basis, when *m* > *n*/2, we can use the isomorphism Gr(*m, n*) ≅ Gr(*n* − *m, n*) to further reduce the computational burden. This will be particularly useful when *m* = *n* − 1 as in Section 4.4. Under this isomorphism Gr(*m, n*) ≅ Gr(*n* − *m, n*), the corresponding subspace of span (*A*) ∈ Gr(*m, n*) is span(*A*_⊥_) ∈ Gr(*n* − *m*, *n*) where *A*_⊥_ is an *n* × (*n* − *m*) matrix such that [*A A*_⊥_] is an orthogonal matrix. Hence the loss function *L*_*u*_ can alternatively be expressed as

$$L_{u}(A,B)=\frac{1}{N}\sum_{i=1}^{N}d^{2}\left(\text{span}\left(X_{i}\right),\text{span}\left(\left(I-A_{⊥}A_{⊥}^{T}\right)X_{i}+A_{⊥}A_{⊥}^{T}B\right)\right).$$

**Remark 1** *The reduction of the space of all possible projections from St*(*m, n*) *to Gr*(*m, n*) *is a consequence of the choice of the loss function L*_*u*_. *With a different loss function, one might have a different space of possible projections*.

### 4.2 Supervised Dimensionality Reduction

If in addition to 𝒳_1_, …, 𝒳_*N*_ ∈ Gr(*p, n*), we are given the associated labels *y*_1_, …*, y*_*N*_ ∈ {1, …*, k*}, then we would like to use this extra information to sharpen the result of dimensionality reduction. Specifically, we expect that after reducing the dimension, points from the same class preserve their proximity while points from different classes are well separated. We use an *affinity function a:Gr(p,n)×Gr(p,n)→ℝ* to encode the structure of the data as suggested by Harandi et al. (2018, Sec 3.1, Eq. (14)-(16)). For completeness, we repeat the definition of the affinity function here. The affinity function is defined as *a*(𝒳_*i*_, 𝒳_*j*_) = *g*_*w*_(𝒳_*i*_, 𝒳_*j*_) − *g*_*b*_(𝒳_*i*_, 𝒳_*j*_) where

$$g_{w}\left({𝒳}_{i},{𝒳}_{j}\right)=\{\begin{array}{ll} 1 & \text{if }{𝒳}_{i}\in N_{w}\left({𝒳}_{j}\right)\text{ or }{𝒳}_{j}\in N_{w}\left({𝒳}_{i}\right)\\ 0 & \text{Otherwise} \end{array}$$

$$g_{b}\left({𝒳}_{i},{𝒳}_{j}\right)=\{\begin{array}{ll} 1 & \text{if }{𝒳}_{i}\in N_{b}\left({𝒳}_{j}\right)\text{ or }{𝒳}_{j}\in N_{b}\left({𝒳}_{i}\right)\\ 0 & \text{Otherwise} \end{array}.$$

The set *Nw*(𝒳_*i*_) consists of *ν*_*w*_ nearest neighbors of 𝒳_*i*_ that have the same labels as *y*_*i*_, and the set *N*_*b*_(𝒳_*i*_) consists of *ν*_*b*_ nearest neighbors of 𝒳_*i*_ that have *different* labels from *y*_*i*_. The nearest neighbors can be computed using the geodesic distance.

The desired projection map *π*_*A*_ that we seek is obtained by the minimizing the following loss function

$$L_{s}(A)=\frac{1}{N^{2}}\sum_{i,j=1}^{N}a\left({𝒳}_{i},{𝒳}_{j}\right)d^{2}\left(\text{span}\left(A^{T}X_{i}\right),\text{span}\left(A^{T}X_{j}\right)\right)$$

where *d* is a distance metric on Gr(*p, m*). Note that if the distance metric *d* has O(*m*)-symmetry, e.g. the geodesic distance, so does *L*_*s*_. In this case the optimization can be done on St(*m, n*)/O(*m*) ≅ Gr(*m, n*). Otherwise it is on St(*m, n*). This supervised dimensionality reduction is termed as, supervised nested Grassmann (sNG).

### 4.3 Choice of the distance function

The loss functions *L*^*u*^ and *L*^*s*^ depend on the choice of the distance $d:\text{Gr}(p,n)\times \text{Gr}(p,n)\to {\mathbb{R}}_{\geq 0}$. Besides the geodesic distance, there are many widely used distances on the Grassmann manifold, see, for example, Edelman et al. (1998, p. 337) and Ye and Lim (2016, Table 2). In this work, we use two different distance metrics: (1) the geodesic distance *d*_*g*_ and (2) the projection distance, which is also called the chordal distance in Ye and Lim (2016) and the projection *F*-norm in Edelman et al. (1998). The geodesic distance was defined in Section 3.1 and the projection distance is defined as follows. For 𝒳, 𝒴 ∈ Gr(*p, n*), denote the projection matrices onto 𝒳 and 𝒴 is given by *p*_𝒳_ and *p*_𝒴_ respectively. Then, the distance between 𝒳 and 𝒴 is given by $d_{p}(𝒳,𝒴)={\left\|P_{𝒳}-P_{𝒴}\right\|}_{F}/\sqrt{2}={\left(\sum_{i=1}^{p}{\text{sin}}^{2}\theta_{i}\right)}^{1/2}$ where *θ*_1_, …, *θ*_*p*_ are the principal angles of 𝒳 and 𝒴. If 𝒳 = span(*X*) then *P*_𝒳_ = *X*(*X*^*T*^ *X*)^−1^ *X*^*T*^. It is also easy to see the the projection distance has O(*n*)-symmetry. We choose the projection distance mainly for its computational efficiency as it involves only matrix multiplication which has a time complexity *O*(*n*^2^) whereas the geodesic distance requires an SVD which has a time complexity of *O*(*n*^3^).

### 4.4 Analysis of Principal Nested Grassmannians

To determine the dimension of the nested submanifold that fits the data well enough, we can choose *p* < *m*_1_ < … < *m*_*k*_ < *n* and estimate the projection onto these nested Grassmann manifolds. The ratio of expressed variance for each projection is the ratio of the variance of the projected data and the variance of the original data. With these ratios, we can choose the desired dimension according to the pre-specified percentage of expressed variance as one would do for choosing the number of principal components in PCA.

Alternatively, one can have a full analysis of principal nested Grassmanns (PNG) as follows. Starting from Gr(*p, n*), one can reduce the dimension down to Gr(*p, p* + 1). Using the diffeomorphism between Gr(*p, n*) and Gr(*p, n* − *p*), we have Gr(*p, p* + 1) ≅ Gr(1*, p* + 1), and hence we can continue reducing the dimension down to Gr(1, 2). The resulting sequence will be

Gr(p,n)→Gr(p,n−1)→⋯→Gr(p,p+1)=Gr(1,p+1)→Gr(1,p)→⋯→Gr(1,2).

Furthermore, we can reduce the points on Gr(1, 2), which is a 1-dimensional manifold, to a 0-dimensional manifold, which is simply a point, by computing the Fréchet mean (FM). We call this FM the nested Grassmannian mean (NGM) of 𝒳_1_, …, 𝒳_*N*_ ∈ Gr(*p, n*). The NGM is unique since $\text{Gr}(1,2)≅\mathbb{R}P^{1}$ can be identified as the half circle in ${\mathbb{R}}^{2}$ and the FM is unique in this case. Note that in general, the NGM will not be the same as the FM of 𝒳_1_, …, 𝒳_*N*_ since the embedding/projection need not be isometric. The supervised PNG (sPNG) can be obtained similarly by replacing each projection with it supervised counterpart.

### 4.5 Principal Scores

**Figure 3:**
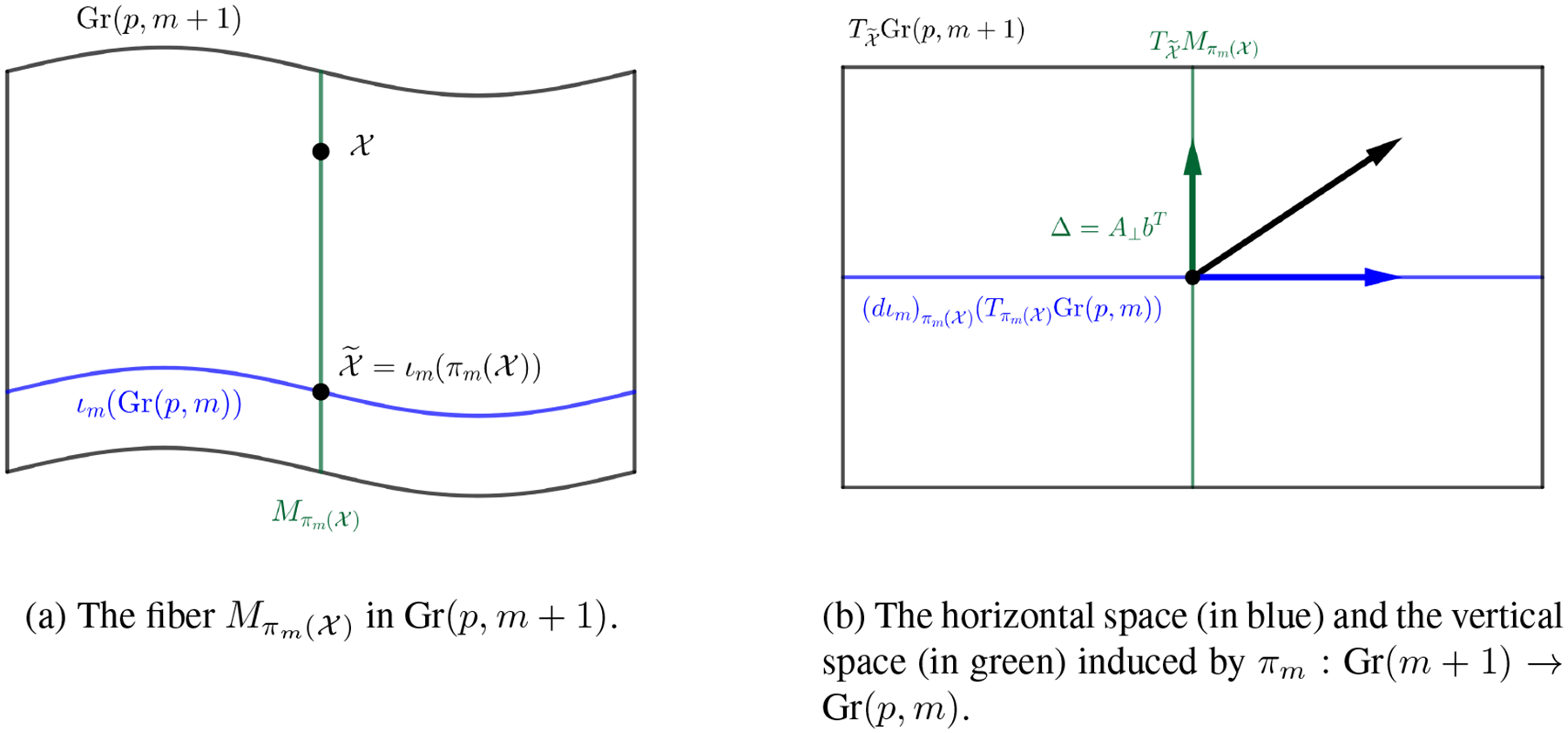
Illustrations of submanifolds induced by *π*_*m*_.

Whenever we apply a projection *π*^*m*^ : Gr(*p*, *m* + 1) → Gr(*p*, *m*) to the data, we might lose some information contained in the data. More specifically, since we project data on a *p*(*m* + 1 − *p*)-dimensional manifold to a *p*(*m* − *p*)-dimensional manifold, we need to describe this *p*(*m* + 1 − *p*) *p*(*m* − *p*) = *p* dimensional information loss during the projection. In PCA, this is done by computing the scores of each principal component, which are the transformed coordinates of each sample in the eigenspace of the covariance matrix. We can generalize the notion of principal scores to the nested Grassmanns as follows: For each 𝒳 ∈ Gr(*p*, *m* + 1), denote by $M_{\pi_{m}(𝒳)}$ the fiber of *π*_*m*_(𝒳), i.e. $M_{\pi_{m}(𝒳)}=\pi_{m}^{-1}\left(\pi_{m}(𝒳)\right)=\left\{𝒴\in \text{Gr}(p,m+1):\pi_{m}(𝒴)=\pi_{m}(𝒳)\right\}$ which is a *p*-dimensional submanifold of Gr(*p*, *m* + 1). An illustration of this fiber is given in Figure 3a. Let $\tilde{𝒳}=\iota_{m}\left(\pi_{m}(𝒳)\right)$ and let the unit tangent vector $V\in T_{\tilde{𝒳}}M_{\pi_{m}(𝒳)}$ be the geodesic direction from $\tilde{𝒳}$ to 𝒳. Given a suitable basis on $T_{\tilde{𝒳}}M_{\pi_{m}(𝒳)}$, *V* can be realized as a p-dimensional vector, and this will be the score vector of 𝒳 associated with the projection, *π*_*m*._

By the definition of $M_{\pi_{m}(𝒳)}$ we have the following decomposition of the tangent space of Gr(*p*, *m* + 1) at $\tilde{𝒳}$ into the horizontal space and the vertical space induced by *π*_*m*_,

$$T_{\tilde{𝒳}}\text{Gr}(p,m+1)=T_{\tilde{𝒳}}M_{\pi_{m}(𝒳)}⊕{\left(d\iota_{m}\right)}_{\pi_{m}(𝒳)}\left(T_{\pi_{m}(𝒳)}\text{Gr}(p,m)\right).$$

An illustration of this decomposition is given in Figure 3b. A tangent vector of $M_{\pi_{m}(𝒳)}$ at $\tilde{𝒳}$ is of the form Δ = *A*_⊥_*b*^*T*^ where *A*_⊥_ is any (m + 1)-dim vector such that [*A A*_⊥_] is orthogonal and $b\in {\mathbb{R}}^{p}$ It is easy to check that *π*_*m*_(span(*AA*^*T*^*X* + A_⊥_b^*T*^)) = *π*_*m*_(span(*X*)) = span(*A*^*T*^*X*). Hence a natural coordinate for the tangent vector $\Delta =A_{⊥}b^{T}$ is $b\in {\mathbb{R}}^{p}$ , and the geodesic direction from $\tilde{𝒳}$ to 𝒳 would be *V*= *X*^*T*^*A*_⊥._ It is easy to see that ∥*V*∥_*F*_ = 1 since *X* has orthonormal columns. To reflect the distance between $\tilde{𝒳}$ and 𝒳, i.e. the reconstruction error, we define $d\left(\tilde{𝒳},𝒳\right)V$ as the score vector for 𝒳 associated with *π*_*m*_. In the case of Gr(1, 2) → NGM, we use the sign distance to the NGM as the score. For complex nested Grassmanns however, the principal score associated with each projection is a p-dimensional complex vector. For the sake of visualization, we transform this *p*-dimensional complex vector to a 2*p*-dimensional real vector. The procedure for computing the PNG and the principal scores is summarized in Algorithm 1.

**Remark 2** *Note that this definition of principal score is not intrinsic as it depends on the choice of basis*. *Indeed*, *it is impossible to choose a p-dimensional vector for the projection π*_*m*_ *in an intrinsic way*, *since the only property of a map that is independent of bases is the rank of the map*. *A reasonable choice of basis is made by viewing the Grassmann Gr*(*p*, *m*) *as a quotient manifold of St*(*p*, *m*), *which is a submanifold in ${\mathbb{R}}^{m\times p}$*. *This is how we define the principal score for nested Grassmanns*.

## 5. Experiments

In this section, we will demonstrate the performance of the proposed dimensionality reduction technique, i.e. PNG and sPNG, via experiments on synthetic and real data. The implementation^1^ is based on the python library pymanopt (Townsend et al., 2016) and we use the steepest descent algorithm for the optimization (with default parameters in pymanopt). The optimization was performed on a desktop with 3.6GHz Intel i7 processors and took about 30 seconds to converge.

### 5.1 Synthetic Data

In this subsection, we compare the performance of the projection and the geodesic distances respectively. The questions we will answer are the following. (1) From Section 4.3, we see that using projection distance is more efficient than using the geodesic distance. But how do they perform compared to each other under varying dimension *n* and variance level *σ*^2^? (2) Is our method of dimensionality reduction ”better” than PGA? Under what conditions does our method outperform PGA?

#### 5.1.1 Projection and Geodesic Distance Comparisons

The procedure we used to generate random points on Gr(*p*, *n*) for the synthetic data experiments is as follows: First, we generate *N* points from a uniform distribution on St(*p*, *m*) (Chikuse, 2003, Ch. 2.5), generate *A* from the uniform distribution on St(*m*, *n*), and generate *B* as an *n* × *p* matrix with i.i.d entries from *N*(0, 0.1). Then we compute ${\tilde{𝒳}}_{i}=\text{span}\left(AX_{i}+\left(I-AA^{T}\right)B\right)\in \text{Gr}(p,n)$. Finally, we compute ${𝒳}_{i}={\text{Exp}}_{{\tilde{𝒳}}_{i}}\left(\sigma U_{i}\right)$, where $U_{i}={\tilde{U}}_{i}/\left\|{\tilde{U}}_{i}\right\|$ and ${\tilde{U}}_{i}\in T_{{\tilde{𝒳}}_{i}}\text{Gr}(p,n)$ to include some perturbation.

**Figure 4:**
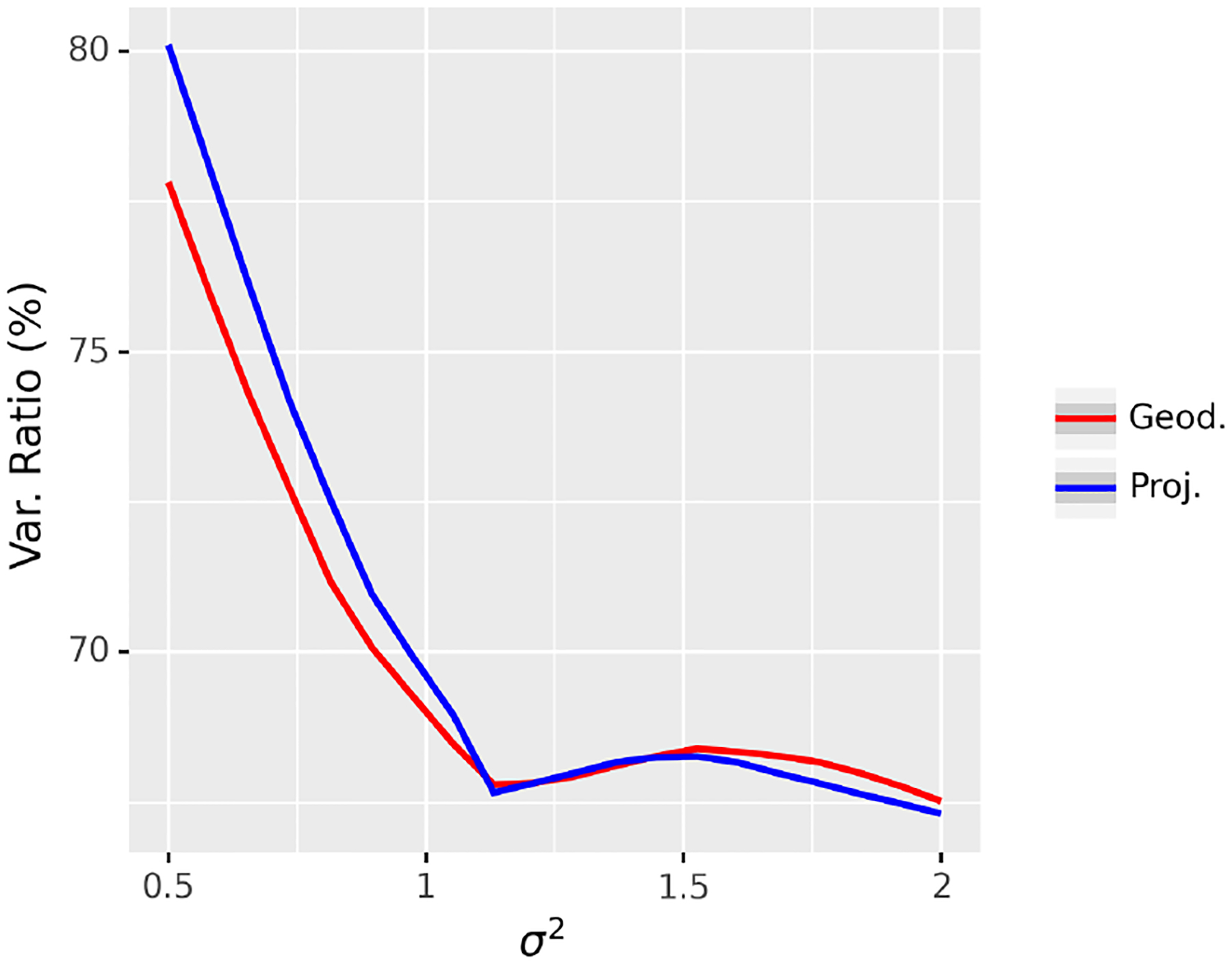
Comparison of the NG representations based on the projection and geodesic distances using the expressed variance.

This experiment involves comparing the performance of the NG representation in terms of the explained variance, under different levels of data variance. In this experiment, we set *N* = 50, *n* = 10, *m* = 3, and *p* = 1 and *σ* is ranging from 0.5 to 1. The results are averaged over 100 repetitions and are shown in Figure 4. From these results, we can see that the explained variance for the projection distance and the geodesic distance are indistinguishable but using projection distance leads to much faster convergence than when using the geodesic distance. The reason is that when two points on the Grassmann manifold are close, the geodesic distance can be well-approximated by the projection distance. When the algorithm converges, the original point 𝒳_*i*_ and the reconstructed point ${\hat{𝒳}}_{i}$ should be close and the geodesic distance can thus be well-approximated by the projection distance. Therefore, for all the experiments in the next section, we use the projection distance for the sake of efficiency.


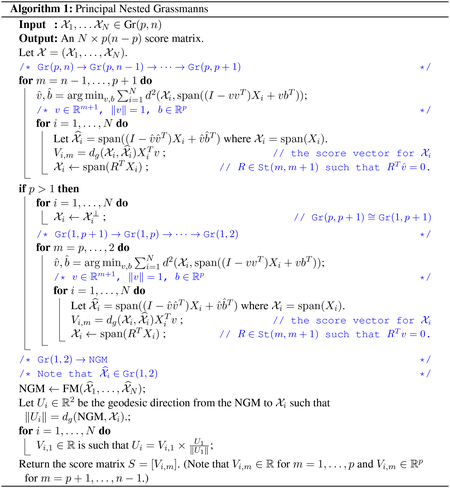


#### 5.1.2 Comparison of PNG and PGA

**Figure 5:**
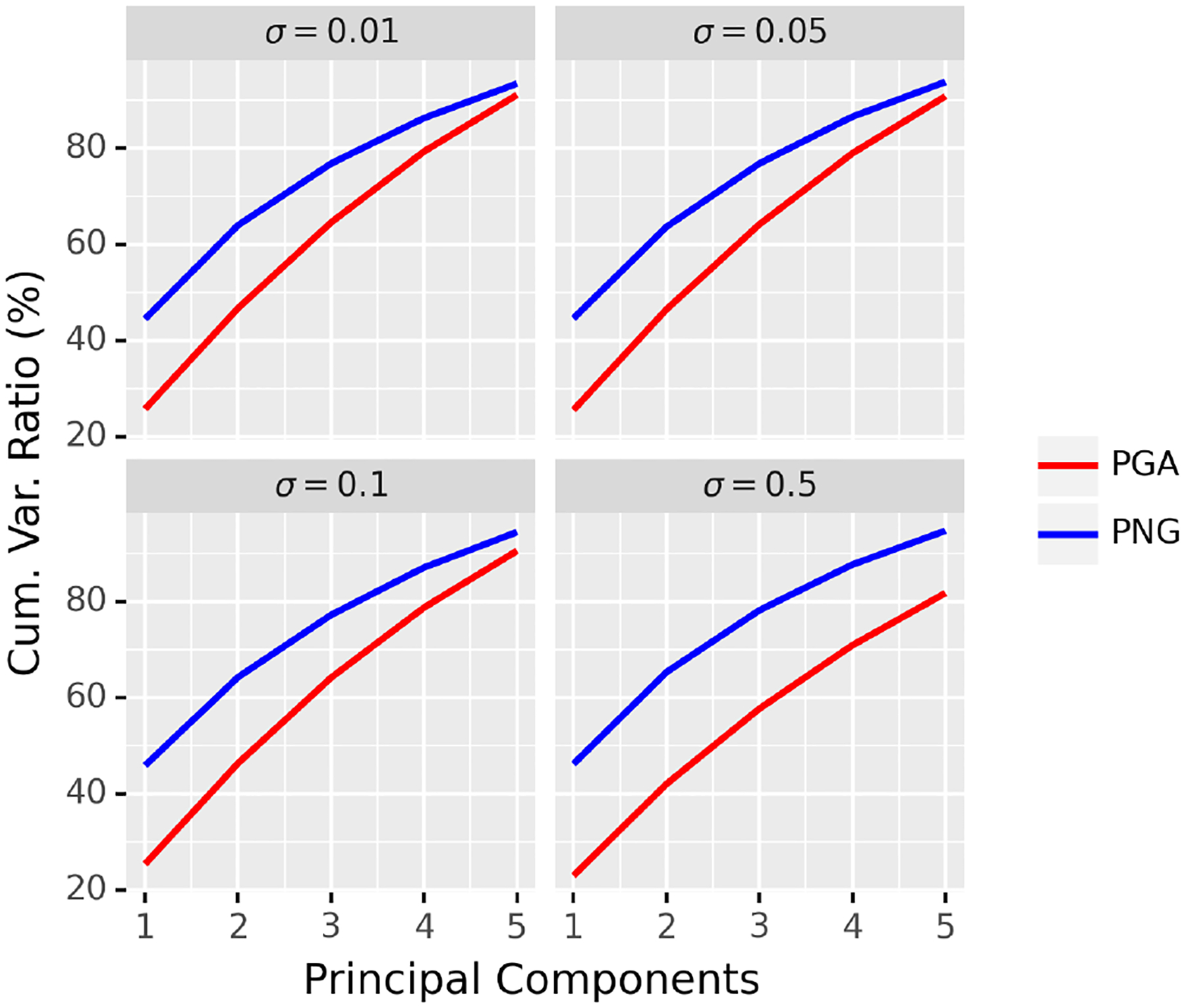
Comparison of PNG and PGA under different levels of noise.

Now we compare the proposed PNG to PGA. In order to have a fair comparison between PNG and PGA, we define the principal components of PNG as the principal components of the scores obtained as in Section 4.5. Similar to the previous experiment, we set *N* = 50, *n* = 10, *m* = 5, *p* = 2, and *σ* = 0.01, 0.05, 0.1, 0.5 and apply the same procedure to generate synthetic data. The results are averaged over 100 repetitions and are shown in Figure 5.

From Figure 5, we can see that our method outperforms PGA by virtue of the fact that it is able to capture a larger amount of variance contained in the data. We can see that when the variance is small, the improvement of PNG over PGA is less significant, whereas, our method is significantly better for the large data variance case (e.g. comparing *σ* = 0.5 and *σ* = 0.01). Note that when the variance in the data is small, i.e. the data are tightly clustered around the FM, and PGA captures the essence of the data well. However, the requirement in PGA on the geodesic submanifold to pass through the anchor point, namely the FM, is not meaningful for data with large variance as explained through the following simple example. Consider, a few data points spread out on the equator of a sphere. The FM in this case is likely to be the north pole of the sphere if we restrict ourselves to the upper hemisphere. Thus, the geodesic submanifold computed by PGA will pass through this FM. However, what is more meaningful is a submanifold corresponding to the equator, which is what a nested spheres representation (Jung et al., 2012) in this case yields. In similar vein, for data with large variance on a Grassmann manifold, our NG representation will yield a more meaningful representation than PGA.

### 5.2 Application to Planar Shape Analysis

We now apply our method to planar (2-dimensional) shape analysis. A planar shape *σ* can be represented as an ordered set of *k* > 2 points in ${\mathbb{R}}^{2}$, called a *k*-ad or a configuration. Here we assume that these *k* points are not all identical. Denote the configuration by *X* which is a *k* × 2 matrix. Let *H* be the (*k* − 1) × *k* Helmert submatrix (Dryden and Mardia, 2016, Ch. 2.5). Then *Z* = *HX*/∥*HX*∥_*F*_ is called the pre-shape of *X* from which the information about translation and scaling is removed. The space of all pre-shapes is called the pre-shape space, denoted by ${𝒮}_{2}^{k}$. By definition, the pre-shape space is a (2*k* − 3)-dimensional sphere. The shape is obtained by removing the rotation from the pre-shape, and thus the shape space is $\Sigma_{2}^{k}={𝒮}_{2}^{k}/\text{O}(2)$. It was shown by Kendall (1984) that $\Sigma_{2}^{k}$ is a smooth manifold and, when equipped with the quotient metric, is isometric to the complex projective space $\mathbb{C}P^{k-2}$ equipped with the Fubini-Study metric (up to a scale factor) which is a special case of the complex Grassmannians, i.e. $\mathbb{C}P^{k-2}≅\text{Gr}\left(1,{\mathbb{C}}^{k-1}\right)$. Hence, we can apply the proposed PNG to planar shapes. For planar shapes, we also compare with the recently proposed principal nested shape spaces (PNSS) (Dryden et al., 2019), which is an application of PNS on the pre-shape space. We will now demonstrate how the PNG performs compared to PGA and PNSS using some simple examples of planar shapes and the OASIS dataset.

#### Examples of Planar Shapes

**Figure 6:**
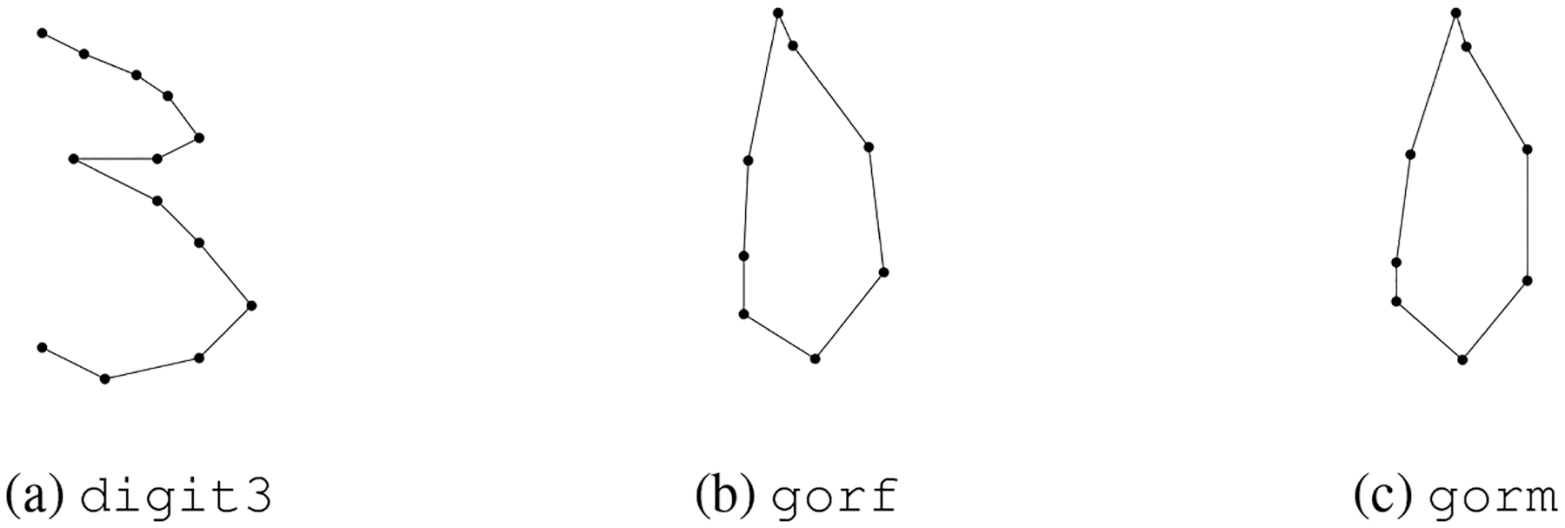
Example shapes from the three datasets.

**Figure 7:**
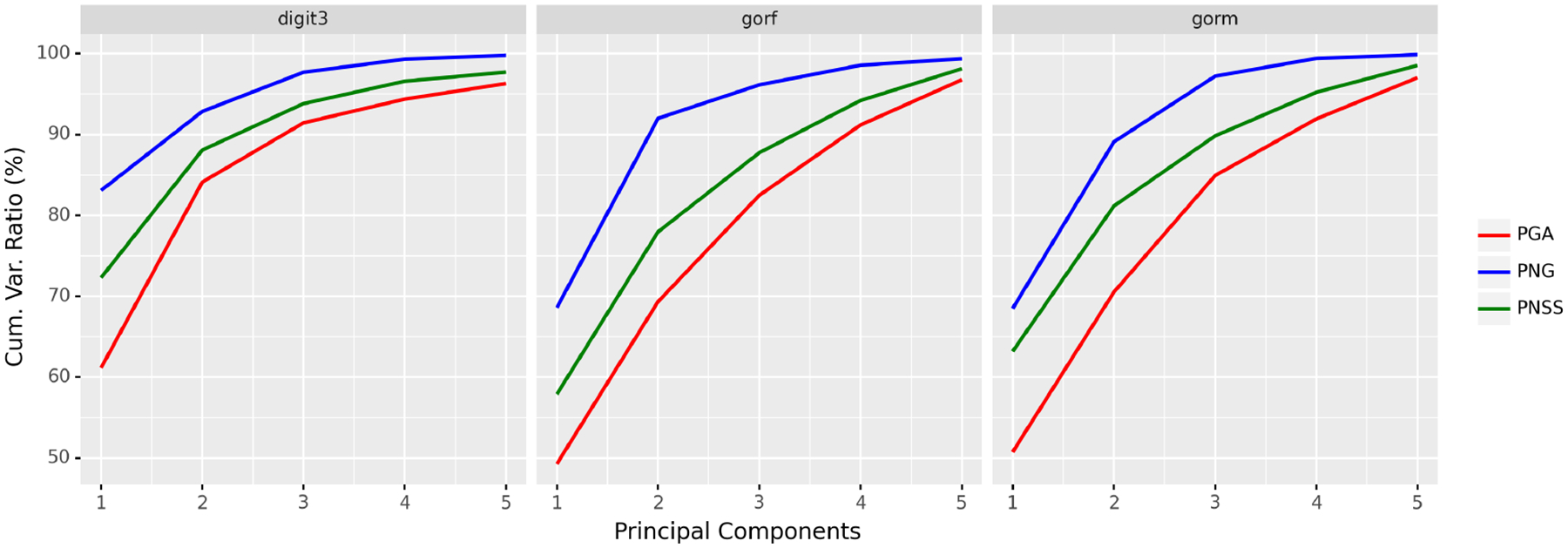
Cumulative explained variance by the first 5 principal components of PNG, PGA, and PNSS.

We take three datasets: digit3, gorf, and gorm, from the R package shapes (Dryden, 2021). The digit3 dataset consists of 30 shapes of the digit 3, each of which is represented by 13 points in ${\mathbb{R}}^{2}$; the gorf dataset consists of 30 shapes of female gorilla skull, each of which is represented by 8 points in ${\mathbb{R}}^{2}$; the gorm dataset consists of 29 shapes of male gorilla skull, each of which is represented by 8 points in ${\mathbb{R}}^{2}$. Example shapes from these three datasets are shown in Figure 6. The cumulative ratios of variance explained by the first 5 principal components^2^ of PNG, PGA, and PNSS are shown in Figure 7. It can be seen from Figure 7 that the proposed PNG achieves higher explained variance than PGA and PNSS respectively in most cases.

#### OASIS Corpus Callosum Data Experiment

**Figure 8:**
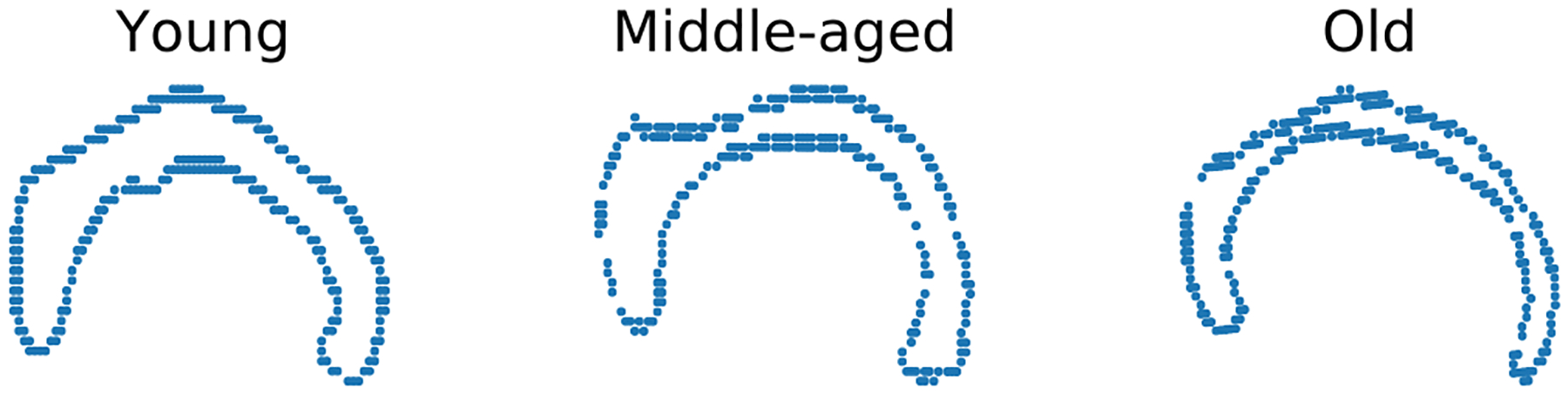
Example Corpus Callosi shapes from three distinct age groups, each depicted using the boundary point sets.

**Figure 9:**
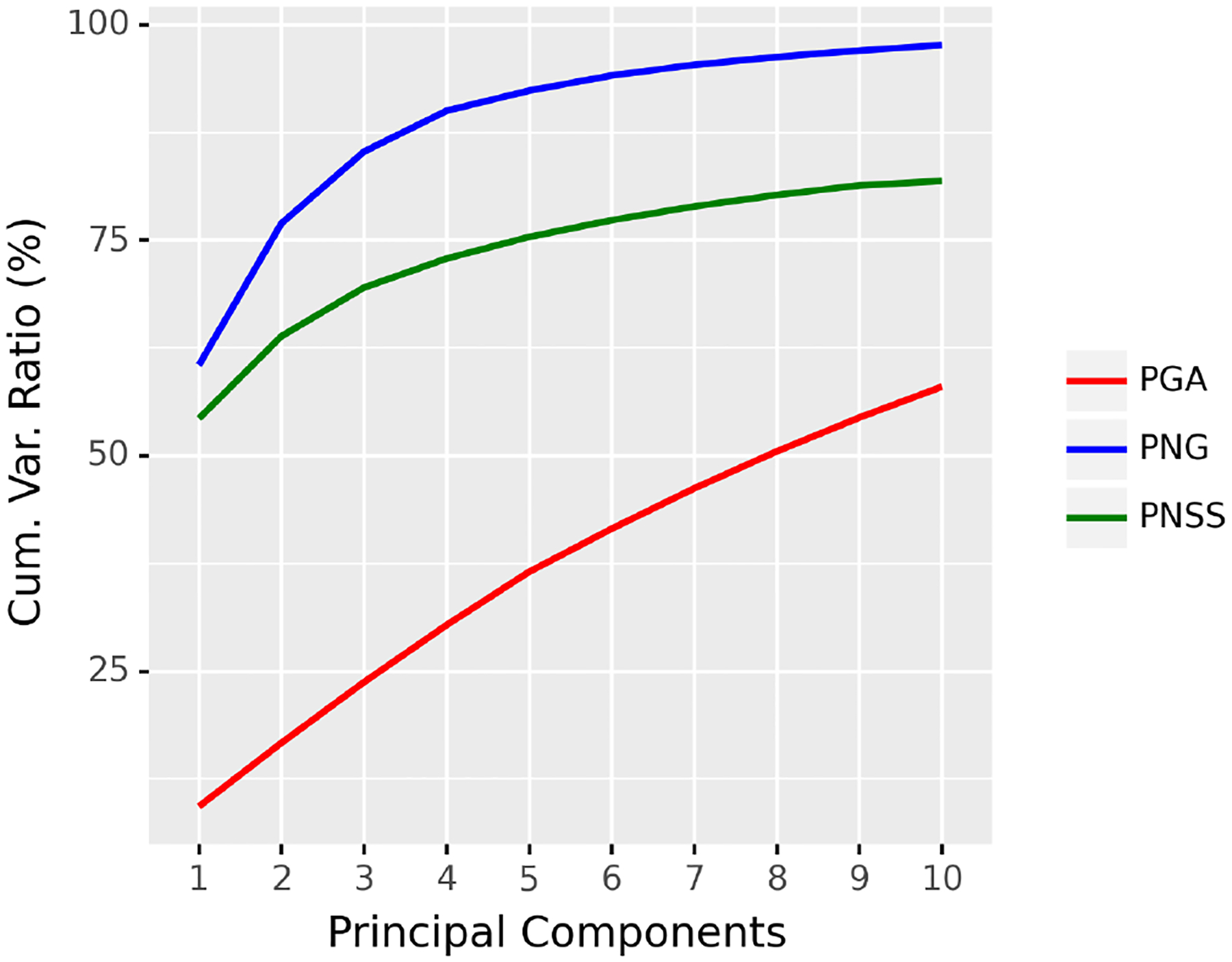
Cumulative explained variance captured by the first 10 principal components of PNG, PGA, and PNSS respectively.

The OASIS database (Marcus et al., 2007) is a publicly available database that contains T1-MR brain scans of subjects of age ranging from 18 to 96. In particular, it includes subjects that are clinically diagnosed with mild to moderate Alzheimer’s disease. We further classify them into three groups: *young* (aged between 10 and 40), *middle-aged* (aged between 40 and 70), and *old* (aged above 70). For demonstration, we randomly choose 4 brain scans within each decade, totalling 36 brain scans. From each scan, the Corpus Callosum (CC) region is segmented and 250 points are taken on the boundary of the CC region. See Figure 8 for samples of the segmented corpus callosi. In this case, the shape space is $\Sigma_{2}^{248}≅\mathbb{C}P^{248}≅\text{Gr}\left(1,{\mathbb{C}}^{249}\right)$. Results of application of the three methods to this data are shown in Figure 9.

Since the data are divided into three groups (young, middle-aged, and old), we can apply the sPNG described in Section 4.2 to reduce the dimension. *The purpose of this experiment is not to demonstrate state-of-the-art classification accuracy for this dataset*. *Instead*, *our goal here is to demonstrate that the proposed nested Grassmann representation in a supervised setting is much more discriminative than the competition*, *namely the supervised PGA*. Hence, we choose a simple classifier such as the support vector machine (SVM) Vapnik (1995) to highlight the aforementioned discriminative power of the nested Grassmann over PGA.

For comparison, the PGA can be easily extended to *supervised PGA* (sPGA) by first diffeomorphically mapping all the data to the tangent space anchored at the FM and then performing supervised PCA Bair et al. (2006); Barshan et al. (2011) on the tangent space. However, generalizing PNSS to the supervised case is nontrivial and is beyond the scope of this paper. Therefore, we limit our comparison to the unsupervised PNSS. In this demonstration, we apply an SVM on the scores obtained from different dimension reduction algorithms, and we choose only the first three principal scores to show that even with the 3-dimensional representation of the original shapes, we can still achieve good classification results. The results are shown in Table 1. These results are in accordance with our expectation since in sPNG, we seek a projection that minimizes the within-group variance while maximizing the between-group variance. However, as we observed earlier, the constraint of requiring the geodesic submanifold to pass through the FM is not well suited for this dataset which has a large variance across the data. This accounts for why the sPNG exhibits far superior performance compared to sPGA in accuracy.

## 6. Conclusion

In this work, we proposed a novel nested geometric structure for homogeneous spaces and used this structure to achieve dimensionality reduction for data residing in Grassmann manifolds. We also discuss how this nested geometric structure served as a natural generalization of other existing nested geometric structures in literature namely, spheres and the manifold of SPD matrices. Specifically, we showed that a lower dimensional Grassmann manifold can be embedded into a higher dimensional Grassmann manifold and via this embedding we constructed a sequence of nested Grassmann manifolds. Compared to the PGA, which is designed for general Riemannian manifolds, the proposed method can capture a higher percentage of data variance after reducing the dimensionality. This is primarily because our method, unlike the PGA, does not require the submanifold to be a geodesic submanifold and to pass through the Fréchet mean of the data. Succinctly, the nested Grassmann structure allows us to fit the data to a larger class of submanifolds than PGA. We also proposed a supervised dimensionality reduction technique which simultaneously differentiates data classes while reducing dimensionality. Efficacy of our method was demonstrated on the OASIS Corpus Callosi data for dimensionality reduction and classification. We showed that our method outperforms the widely used PGA and the recently proposed PNSS by a large margin.

## Figures and Tables

**Table 1:** Classification accuracies for sPGA and sPNG respectively.

Method	Accuracy
sPNG	83.33%
PNG	75%
sPGA	66.67%
PGA	63.89%
PNSS	80.56%

## Footnotes

1. Our code is available at https://github.com/cvgmi/NestedGrassmann.

2. Here the principal components in PNG and PGA are complex whereas the principal components in PNSS are real. Hence, we choose 5 principal components in PNG and PGA and 10 principal components in PNSS, so that the reduced (real) dimension is 10 in all three cases.
